# Augmentation of Antitumor Immunity by Fusions of Ethanol-Treated Tumor Cells and Dendritic Cells Stimulated via Dual TLRs through TGF-β1 Blockade and IL-12p70 Production

**DOI:** 10.1371/journal.pone.0063498

**Published:** 2013-05-24

**Authors:** Shigeo Koido, Sadamu Homma, Masato Okamoto, Yoshihisa Namiki, Kazuki Takakura, Akitaka Takahara, Shunichi Odahara, Shintaro Tsukinaga, Toyokazu Yukawa, Jimi Mitobe, Hiroshi Matsudaira, Keisuke Nagatsuma, Mikio Kajihara, Kan Uchiyama, Seiji Arihiro, Hiroo Imazu, Hiroshi Arakawa, Shin Kan, Kazumi Hayashi, Hideo Komita, Yuko Kamata, Masaki Ito, Eiichi Hara, Toshifumi Ohkusa, Jianlin Gong, Hisao Tajiri

**Affiliations:** 1 Division of Gastroenterology and Hepatology, Department of Internal Medicine, The Jikei University School of Medicine, Tokyo, Japan; 2 Institute of Clinical Medicine and Research, The Jikei University School of Medicine, Chiba, Japan; 3 Department of Oncology, The Jikei University School of Medicine, Tokyo, Japan; 4 Division of Cellular Signaling, Institute for Advanced Medical Research, Keio University School of Medicine, Tokyo, Japan; 5 Department of Endoscopy, The Jikei University School of Medicine, Tokyo, Japan; 6 Institute for Clinical Oncology, Saitama Cancer Center Research, Saitama, Japan; 7 Department of Medicine, Boston University School of Medicine, Boston, Massachusetts, United States of America; University of Bergen, Norway

## Abstract

The therapeutic efficacy of fusion cell (FC)-based cancer vaccine generated with whole tumor cells and dendritic cells (DCs) requires the improved immunogenicity of both cells. Treatment of whole tumor cells with ethanol resulted in blockade of immune-suppressive soluble factors such as transforming growth factor (TGF)-β1, vascular endothelial growth factor, and IL-10 without decreased expression of major histocompatibility complex (MHC) class I and the MUC1 tumor-associated antigen. Moreover, the ethanol-treated tumor cells expressed “eat-me” signals such as calreticulin (CRT) on the cell surface and released immunostimulatory factors such as heat shock protein (HSP)90α and high-mobility group box 1 (HMGB1). A dual stimulation of protein-bound polysaccharides isolated from *Coriolus versicolor* (TLR2 agonist) and penicillin-inactivated *Streptococcus pyogenes* (TLR4 agonist) led human monocyte-derived DCs to produce HSP90α and multiple cytokines such as IL-12p70 and IL-10. Interestingly, incorporating ethanol-treated tumor cells and TLRs-stimulated DCs during the fusion process promoted fusion efficiency and up-regulated MHC class II molecules on a per fusion basis. Moreover, fusions of ethanol-treated tumor cells and dual TLRs-stimulated DCs (E-tumor/FCs) inhibited the production of multiple immune-suppressive soluble factors including TGF-β1 and up-regulated the production of IL-12p70 and HSP90α. Most importantly, E-tumor/FCs activated T cells capable of producing high levels of IFN-γ, resulting in augmented MUC1-specific CTL induction. Collectively, our results illustrate the synergy between ethanol-treated whole tumor cells and dual TLRs-stimulated DCs in inducing augmented CTL responses *in vitro* by FC preparations. The alternative system is simple and may provide a platform for adoptive immunotherapy.

## Introduction

It is well accepted that dendritic cells (DCs) are potent antigen-presenting cells (APCs) that have been used in cancer vaccines because of their ability to initiate cytotoxic T lymphocyte (CTL)-mediated immune responses [1]. Therefore, different strategies have been developed to load DCs with tumor antigens, tumor RNA, tumor lysates, and apoptotic tumor cells [2]–[5]. An alternative strategy for inducing antitumor immunity is the use of fusion cells (FCs) derived from whole tumor cells and DCs. In this approach, tumor-associated antigens (TAAs), both known and unidentified, can be delivered to DCs, processed, and presented through both major histocompatibility complex (MHC) class I and class II pathways [6]. Another advantage of a FC strategy is that modifications to whole tumor cells and DCs can be performed independently while their characters persist after fusion. Therefore, the therapeutic efficacy of FC requires the improved immunogenicity of both whole tumor cells and DCs.

Many tumor cells secrete multiple immune-suppressive factors such as transforming growth factor β1 (TGF-β1), vascular endothelial growth factor (VEGF), and IL-10. Thus, the environment of whole tumor cells used for a FC strategy also has to be modified to become stimulatory immunogenic. Effective adjuvants for generating immunogenic whole tumor cells are stressed molecules to which the ability of apoptotic and necrotic tumor cells has been attributed [7], [8]. In this study, we designed a simple and rapid strategy for reprogramming the immune-suppressive nature of tumor cells by ethanol-treatment. The ethanol-treated tumor cells expressed “eat-me” signals on the cell surface such as calreticulin (CRT) and released immunostimulatory factors such as heat shock protein (HSP)90α and high-mobility group box 1 (HMGB1).

One of the most effective adjuvants for DC activation are Toll-like receptors (TLRs) that have recently emerged as key receptors responsible for recognizing specific conserved components of microbes [9]. Full activation of DCs requires the assembly of receptor signaling complexes by combined TLR agonists [10], thus, we used both protein-bound polysaccharides isolated from *Coriolus versicolor* (PSK; TLR2 agonist) and penicillin-inactivated *Streptococcus pyogenes* (OK-432; TLR4 agonist). Both PSK and OK-432 are good manufacturing practice (GMP) grade agents have been used clinically [11], [12], as they have the capacity to stimulate DCs, T cells, and natural killer (NK) cells [13]–[15]. A dual stimulation of TLR agonists led human monocyte-derived DCs to produce HSP90α and multiple cytokines such as IL-12p70 and IL-10.

We have demonstrated that fusions of ethanol-treated tumor cells and DCs stimulated via dual TLRs are highly immunogenic and induce augmented antigen-specific CTL responses *in vitro* through TGF-β1 blockade and IL-12p70 production.

## Materials and Methods

### Tumor Cells and Conditioned Medium

The human pancreatic cancer cell line (HLA-A*0201), PANC-1 was purchased from American Type Culture Collection (ATCC, Manassas, VA). The human TGF-β1 coding region was cloned from pCMV-SPORT6 (Open Biosystems, Lafayette, CO) and the fragment was inserted to a *Nhe*I*/Hin*dIII site of pcDNA3.1(+). The constructed plasmid was transfected to PANC-1 cells (PANC/TGF-β) using Lipofectamine 2000 transfection reagent (Life Technologies, Tokyo, Japan) and selected with 1 mg/mL geneticin (G418; Life Technologies). PANC-1 cells stably transfected to express empty empty vector (PANC/Mock) were also generated. Cells were maintained in DMEM supplemented with 100 U/mL penicillin, 100 µg/mL streptomycin and 10% fetal calf serum (FCS). Next, to establish FCS-independent PANC/Mock, PANC/TGF-β, and PANC-1 cells, the tumor cell lines were cultured in TIL Media I medium (IBL, Gunma-ken, Japan) containing 10% human plasma protein fraction (PPF) (Baxter Healthcare Corp., Tokyo, Japan) [16]. Treatment of tumor cells with ethanol was achieved by adding 20% (v) pharmaceutical grade ethanol for 5, 10, or 15 min on ice. Conditioned medium from 20% (v) ethanol-treated (15 min on ice) or untreated tumor cells (1×10^4^ cells/mL/well) without geneticin were collected after 48 h culture, centrifuged to remove cells, and stored until use at −80°C.

### Generation of Monocyte-derived DCs

The study protocol was reviewed and approved by the ethics committee of the Jikei Institutional Review Board, Jikei University School of Medicine, as well as the clinical study committee of the Jikei University Kashiwa hospital (No. 14–60 (3209)). Peripheral blood mononuclear cells (PBMCs) from whole blood were obtained with individual written informed consent. Monocyte-derived DCs from healthy HLA-A*0201 donors were generated essentially as described previously [17]. In brief, PBMCs were prepared by Ficoll density-gradient centrifugation and incubated in tissue culture flasks at 37°C for 30 min in AIM V (Life Technologies Japan Ltd.) supplemented with 1% heat-inactivated autologous serum. After incubation for 60 min at 37°C to allow for adherence, nonadherent cells were removed, and adherent cells were cultured for 4 days in AIM V supplemented with 1% heat-inactivated autologous serum, 1000 U/mL GM-CSF (PeproTech, Rocky Hill, NJ) and 500 U/mL IL-4 (Diaclone Research, Boulevard Fleming, France) to generate immature DCs (Imm-DCs). On day 4, Imm-DCs were stimulated with 0.1 KE/mL (0.1 KE equals 10 µg of dried *streptococci*) OK-432 (Chugai Pharmaceutical, Tokyo, Japan) and 100 µg/mL PSK (Kureha Corp., Tokyo, Japan) (TLRs-DCs) for additional 2 days. TLRs-DCs were also generated with the addition of 20% (v) medium conditioned harvested from tumor cells from day 0 onward.

### Generation of FC Preparations

We developed four types of FC preparation by alternating tumor cells as follows: TLRs-DCs fused with PANC/Mock (Mock/FCs), PANC/TGF-β (TGF-β/FCs), ethanol-treated PANC/Mock (E-Mock/FCs), or ethanol-treated PANC/TGF-β (E-TGF-β/FCs). Treatment of tumor cells with ethanol was achieved by adding 20% (v) pharmaceutical grade ethanol for 15 min on ice, washed three times, and immediately used for fusions with TLRs-DCs. Briefly, tumor cells (HLA-A*0201) and TLRs-DCs (HLA-A*0201) were mixed at a ratio of 1∶10 and fusions were generated using 50% polyethylene glycol (PEG) (Sigma-Aldrich, St Louis, MO) [17]. FC preparations were maintained in AIM V supplemented with 1000 U/mL GM-CSF, 500 U/mL IL-4, 0.1 KE/mL OK-432, 100 µg/mL PSK, and 10% PPF. After 2 days culture, FC preparations were integrated to a single entity and purified by gentle pipetting [17].

### Phenotype Analysis

Cells were incubated with FITC-conjugated monoclonal antibodies (mAbs) against MUC1 (HMPV; BD Pharmingen, San Jose, CA), MHC class I (W6/32), MHC class II (HLA-DR), B7-1 (CD80), B7-2 (CD86), TLR2 (CD282), TLR4 (CD284) (BD Pharmingen), HLA-A2 (One Lambda, Canoga Park, CA), CRT (FMC75; Abcam, Cambridge, MA), CD31 (WM59; BD Pharmingen), CD47 (B6H12; eBioscience, San Diego, CA), or matched isotype control IgG. For DC phenotype, DC populations were gated based on their forward- *vs.* side-scatter profile then analyzed for expression of MHC class I, MHC class II, CD80, CD86, CD83, and CCR7. For analysis of ethanol-induced apoptosis and necrosis, untreated and ethanol-treated tumor cells were cultured for 48 h and evaluated by FITC-Annexin V binding assay and Propidium Iodide (PI) (BD Pharmingen). For most accurate analysis of cell changes, the entire scatter population (excluding obvious debris) was gated. Annexin V vs PI plots from the gated cells show the populations corresponding to viable and non-apoptotic (Annexin V^−^PI^−^), early (Annexin V^+^PI^−^), and late (Annexin V^+^PI^+^) apoptotic and necrotic cells. For dual expression in FCs, incubation was performed with FITC-conjugated mAbs against MUC1 and PE-conjugated mAbs against HLA-DR. After the cell aggregations were gated out [17], FC was determined by FACS analysis, where the fused cells were identified as MUC1^+^HLA-DR^+^. Cells were fixed with 2% paraformaldehyde, and analyzed by BD FACSCalibur flow cytometer (Beckton Dickinson, Mountain View, CA) using FlowJo analysis software (Tree Star, OR, USA).

### T cell Stimulation

The number of FC was described based on the number of cells that coexpressed HLA-DR and MUC1 in the FC preparations. Equal numbers of FC preparations (HLA-A*0201) were cocultured with nonadherent PBMCs (HLA-A*0201) at a ratio of 1∶10 in the absence of recombinant human (rh)IL-2 for 3 days. To stimulate and proliferate antigen-specific T cells, a low dose of rhIL-2 (10 U/mL; Shionogi, Osaka, Japan) was added to the culture on day 4. On day 7, T cells were purified after being passed through nylon wool to remove APCs. DCs alone, tumor cells alone, and DCs mixed with tumor cells were used as controls.

### Enzyme-linked Immunosorbent Assay (ELISA)

To assess the production of IL-12p70, VEGF, IL-10, TGF-β1 (R&D Systems), HSP90α (Enzo Life Sciences, Farmingdale, NY), or HMGB1 (Shino-Test, Tokyo, Japan) in FC preparations (1×10^5^ cells/mL/well), DCs (1×10^5^ cells/mL/well), and tumor cells (1×10^4^ cells/mL/well), these cells were cultured in 48-well plates for 48 h at 37°C. Supernatants from these cells were collected and tested for IL-12p70, VEGF, IL-10, TGF-β1, HSP90α, HMGB1 by ELISA according to manufacture’s instructions. To measure the total (latent and active) amount of TGF-β1, the latent form was converted to the active form by treatment with hydrochloric acid. The active form of TGF-β1 was analyzed directly. To critically assess the stimulating ability of T cells by FCs in the induction phase, T cells (1×10^6^ cells/2 mL/well) were cocultured with FCs or an unfused mixture of DCs and tumor cells at a ratio of 10∶1 in the absence of rhIL-2 for 2 days. On day 2, supernatants from these samples were tested for IFN-γ (R&D Systems) immediately, by ELISA according to manufacture’s instructions. The background cytokine levels in conditioned medium were subtracted from each sample.

### Pentamer Staining

Pentamer assays of soluble class I MHC-peptide complexes were used to detect MUC1-specific CTL activity restricted by HLA-A2. Complexes of PE-labeled HLA-A2-MUC1 pentamer (STAPPVHNV) or irrelevant pentamer (Proimmune, Oxford, UK) were used. Briefly, stimulated T cells were incubated with PE-conjugated MUC1 pentamer for 1 h at 4°C. After washing, the T cells were stained with FITC-conjugated mAbs against CD8, washed, fixed with 2% paraformaldehyde, and analyzed by flow cytometry using FlowJo analysis software. Complexes of PE-irrelevant pentamers were used as controls. CD8^+^ T cell reactivity to MUC1 is shown as the percentage of the total population of CD8^+^ T cells that were double positive (CD8^+^MUC1 pentamer^+^).

### Cytotoxicity Assays

The cytotoxicity assays were performed by flow cytometric analysis using Active Caspase-3 Apoptosis kit I (BD Pharmingen) that measured CTL-induced caspase-3 activation in target cells by detecting the specific cleavage of fluorogenic caspase-3 [18]. PANC-1 cells were labeled with PKH-26 (Sigma-Aldrich), washed, cultured with stimulated T cells for 2 h at 37°C in 96-well, V-bottomed plates at the indicated effector cell:T cell (E:T) ratios. Cells were fixed with Cytofix/Cytoperm Solution (BD Pharmingen), washed with Perm/Wash Buffer (BD Pharmingen), and incubated with FITC-conjugated mAbs against human active caspase-3 substrate (BD Pharmingen) for 30 min at room temperature, followed by two washes with Perm/Wash buffer. In certain experiments, PANC-1 cells were preincubated with anti-HLA-A2 (One Lambda) or control IgG for 30 min at 37°C before the addition of effector cells. The percentage of cytotoxicity (mean ± SD of three replications) was determined with the following equation: percentage of caspase-3 staining = (caspase-3^+^PKH-26^+^ cells)/(caspase-3^+^PKH-26^+^ cells+caspase-3^−^PKH-26^+^ cells) × 100.

### Statistical Analysis

Results are expressed as mean ± SD as indicated in the legends. We used one-way analysis of variance to determine significance. When P-values were 0.05 or less, differences were considered statistically significant.

## Results

### Characterization of Tumor Cells

**Figure 1:**
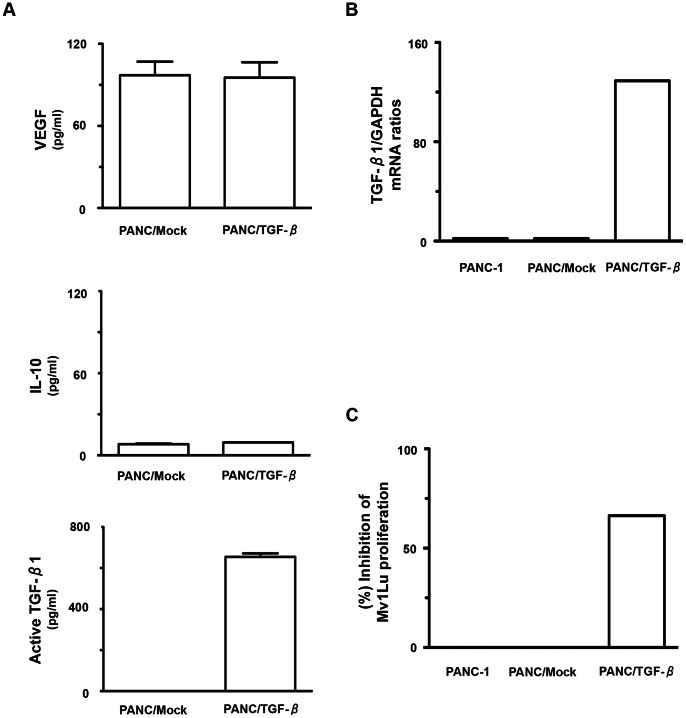
Characterization of a stable transfectant of PANC-1 cells expressing TGF-β1. (A) Mean concentration of VEGF, IL-10, the active form of TGF-β1 derived from PANC/Mock or PANC/TGF-β was analyzed by ELISA. (B) TGF-β1 mRNA levels from PANC-1/Mock, PANC/TGF-β, or PANC-1 cells was examined using real-time PCR. (C) The effects of active TGF-β1 from PANC-1/Mock, PANC/TGF-β, or PANC-1 cells in the supernatants on Mv1Lu cells were analyzed.

This study was designed to assess the role of multiple immune-suppressive cytokines including TGF-β1 derived from tumor cells, which were used for FC preparations on the induction of MUC1-specific CTLs restricted by HLA-A2. Thus, we used the MUC1 and HLA-A2 positive tumor cell line, PANC-1. Considerable quantities of VEGF, low levels of IL-10 and little or none of the active form of TGF-β1 (Fig. 1A), but not IL-12p70 (data not shown), were detected in culture supernatants of PANC-1 cells. To critically assess the effects of tumor-derived TGF-β1 on DCs and FCs, we generated a stable transfectant of PANC-1 cells expressing high levels of TGF-β1 (PANC/TGF-β). The TGF-β1 mRNA expression level in PANC/TGF-β1 was much higher than in the cell line transfected with the expression vector alone (PANC/Mock) as well as untransfected PANC-1 cells (Fig. 1B). While the active form of TGF-β1 was produced at high levels in PANC/β that could inhibit Mv1Lu cell growth, the supernatants from PANC/Mock as well as PANC-1 cells did not (Fig. 1A and C). In addition, there was no difference in the production of VEGF and IL-10 between PANC/TGF-β, PANC/Mock, and PANC-1 cells (data not shown).

### Characterization of Ethanol-treated Tumor Cells

**Figure 2:**
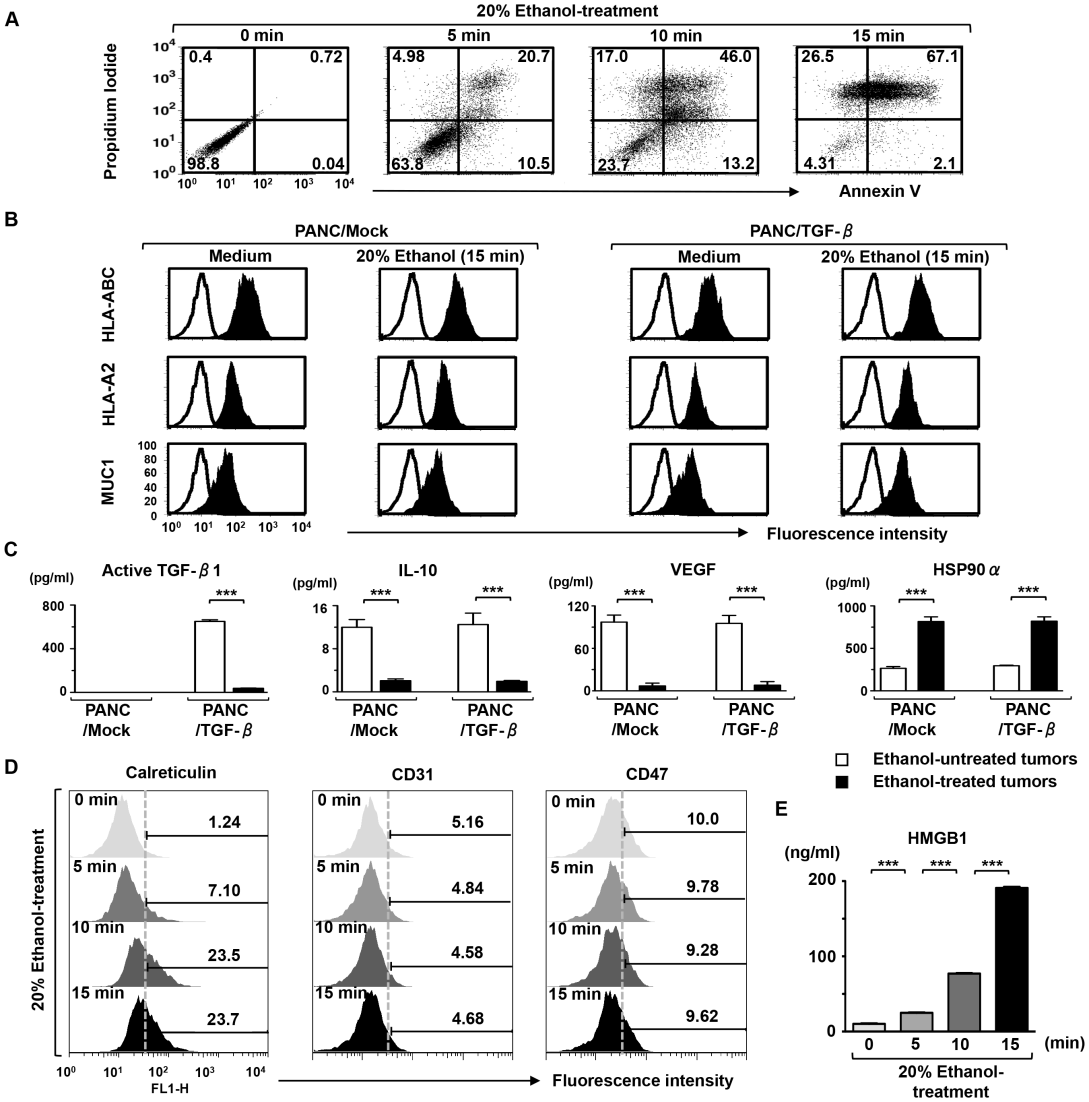
Phenotypic and functional characterization of ethanol-treated tumor cells. (A) PANC/TGF-β were treated with 20% ethanol for 5, 10, or 15 min on ice and cultured for 48 h at 37°C in a 5% CO_2_ humidified atmosphere. Ethanol-treated PANC/TGF-β were stained with FITC-conjugated mAbs against Annexin V and propidium iodide (PI). The entire scatter population (excluding obvious debris) was gated for most accurate analysis of cell changes. Cells undergoing early apoptosis are Annexin V positive and PI negative, and cells in late apoptosis or already dead (apoptotic death and necrotic death) are Annexin V and PI positive. (B) Ethanol-treated (20%, 15 min, on ice) or untreated tumor cells (PANC/Mock and PANC/TGF-β) were analyzed by flow cytometry for expression of HLA-ABC, HLA-A2 and MUC1 (solid area). The entire scatter population (excluding obvious debris) was gated. Unfilled histogram profile indicates the isotype control, and solid histogram indicates the specific antibody. (C) Mean concentration of the active form of TGF-β1, IL-10, VEGF, and HSP90α by ethanol-treated (▪) and untreated (□) tumor cells (PANC/Mock and PANC/TGF-β) (1×10^4^/mL) was analyzed by ELISA. (D) PANC/TGF-β were treated with 20% ethanol for 5, 10, or 15 min on ice and cultured for 48 h at 37°C in a 5% CO_2_ humidified atmosphere. The entire scatter population (excluding obvious debris) was gated. Ethanol-treated and untreated PANC/TGF-β were analyzed by flow cytometry for expression of calreticulin (left panel), CD31 (middle panel), and CD47 (right panel). Untreated PANC/TGF-β were used as controls. Solid histogram indicates the specific antibody. (E) Mean concentration of HMGB1 by PANC/TGF-β treated with 20% ethanol for 5, 10, or 15 min on ice or untreated PANC/TGF-β (1×10^5^/mL) was analyzed by ELISA. The mean ± SD of three experiments is shown. ***P<0.001; **P<0.01; *P<0.05.

Treatment of PANC/TGF-β (Fig. 2A), PANC/Mock, and PANC-1 cells (data not shown) with 20% (v) ethanol for 15 min on ice resulted in the inhibition of cell growth and sub-lethal aggression as shown by the induction of apoptosis and necrosis. In addition, the majority of untreated tumor cells were non-apoptotic and necrotic (Annexin V^−^PI^−^) (Fig. 2A). Although the ethanol-treated tumor cells maintained their cell shape and size, higher concentrations of ethanol caused deformation of the cells and was lethal (data not shown). The ethanol-treated and untreated tumor cells (PANC/Mock and PANC/TGF-β) showed sustained expression of HLA-ABC, HLA-A2, MUC1 (Fig. 2B), TLR2, and TLR4, but not HLA-DR, CD80, CD86, and CD83 (data not shown). In addition, there was no difference in phenotype between PANC/Mock and PANC-1 cells, whether ethanol-treated or not (data not shown).

Next, to assess the immunogenicity of ethanol-treated tumor cells, the production of immune-suppressive cytokines such as the active form of TGF-β1, VEGF, and IL-10 in tumor cells was analyzed. Although PANC/TGF-β produced high levels of the active form of TGF-β1, treatment of PANC/TGF-β with 20% (v) ethanol for 15 min on ice, resulted in a marked inhibition of TGF-β1 production (Fig. 2C). Moreover, VEGF and IL-10 production was also inhibited by ethanol-treatment (Fig. 2C). Interestingly, treatment of the tumor cells (PANC/Mock and PANC/TGF-β) with ethanol enhanced production of extracellular HSP90α (Fig. 2C). When ethanol was used at 5% (v) or less, no effect on HSP90α expression was observed (data not shown). The ethanol-treated PANC/β also expressed increased levels of cell surface molecules that can stimulate phagocytosis, such as CRT and HMGB1 (Fig. 2D and E), but not inhibitory phagocytosis molecules, such as CD47 and CD31 (Fig. 2D). In addition, there was no difference in the expression of the active form of TGF-β1, VEGF, IL-10, extracellular HSP90α, CRT, HMGB1, CD47, and CD31 between the three types of ethanol-treated tumor cells (PANC/Mock, PANC/TGF-β, and PANC-1) (data not shown).

### Characterization of DCs Stimulated via TLRs

**Figure 3:**
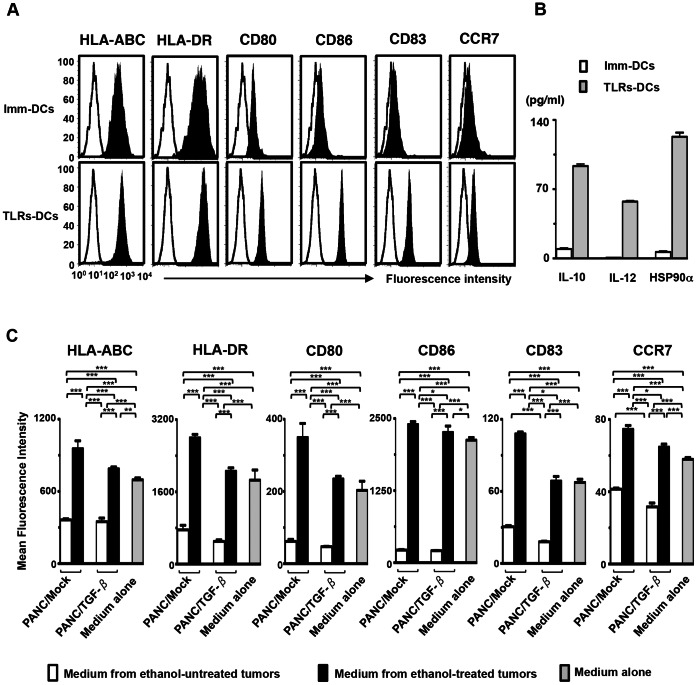
Phenotypic and functional characterization of DCs. (A) Imm-DCs and TLRs-DCs populations were gated based on their forward- *vs.* side-scatter profile then analyzed for expression of the indicated antigens (solid area) (n = 5). Unfilled histogram profile indicates the isotype control, and solid histogram indicates the specific antibody. (B) Mean concentration of IL-10, IL-12, or HSP90α by Imm-DCs and TLRs-DCs (1×10^5^/mL) was analyzed by ELISA (n = 5). (C) TLRs-DCs were generated in conditioned medium derived from ethanol-treated, untreated tumor cells (PANC/Mock or PANC/TGF-β), or control cultures without supernatants from tumor cells (medium alone) (n = 4). DC populations were gated based on their forward- *vs.* side-scatter profile then analyzed for expression of MHC class I, MHC class II, CD80, CD86, CD83, CCR7, or matched isotype IgG control. MFI of HLA-ABC, HLA-DR, CD80, CD86, CD83, and CCR7 was analyzed. The results are expressed as the mean ± SD. ***P<0.001; **P<0.01; *P<0.05.

Although Imm-DCs displayed a characteristic phenotype with the expression of HLA-ABC, HLA-DR, CD80, and CD86, but low levels of CD83 and CCR7 [19], TLRs-DCs displayed a characteristic phenotype with the high expression of HLA-ABC, HLA-DR, CD80, and CD86, CD83, and CCR7 (Fig. 3A). Moreover, TLRs-DCs exhibited increased production of IL-12p70, IL-10, and extracellular HSP90α compared with Imm-DCs (Fig. 3B). In addition, the production of active TGF-β1 and VEGF from either type of DC was base line (data not shown). These results suggest that TLRs-DCs are more active than Imm-DCs.

### Effects of Immune-suppressive Cytokines Secreted by Tumor Cells on TLRs-DCs

The concurrent production of multiple immune-suppressive cytokines by whole tumor cells raised the issue of how a combination of these cytokines would affect the activation state of DCs. To address this issue, TLRs-DCs were generated with or without conditioned supernatants from tumor cells. The supernatants derived from PANC/Mock affected the phenotype of TLRs-DCs, as demonstrated by the decreased mean fluorescence intensity (MFI) of HLA-ABC, HLA-DR, CD80, CD86, CD83, and CCR7 (Fig. 3C). Notably, generation of DCs with supernatants derived from PANC/TGF-β resulted in an inactive phenotype, even if DCs were stimulated with combined OK-432 and PSK (Fig. 3C). Interestingly, supernatants derived from the ethanol-treated tumor cells (PANC/Mock or PANC/TGF-β) induced active TLRs-DC phenotypes compared with those generated with supernatants from ethanol-untreated tumor cells or medium alone (Fig. 3C). Moreover, generation of TLRs-DCs with supernatants from ethanol-treated tumor cells exhibited characteristic morphology with multiple fine dendrites (data not shown). In addition, there was no difference in TLRs-DC phenotypes generated with supernatants derived from PANC-1 cells compared with that generated with PANC/Mock (data not shown).

### Effects of Immune-suppressive Cytokines Secreted by Tumor Cells on FC Phenotypes

**Figure 4:**
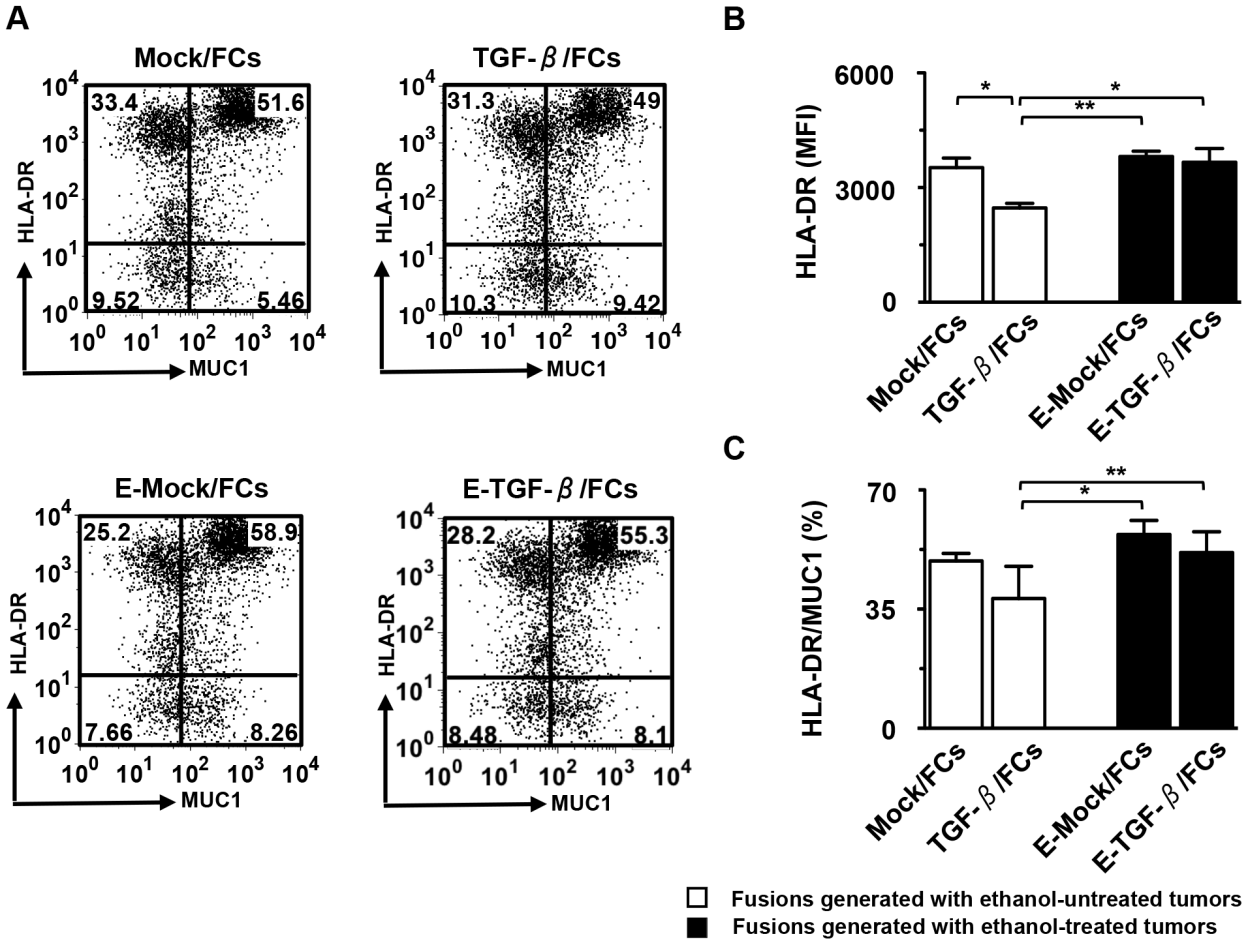
Phenotypic characterization of FC preparations. (A) Four types of FC preparations (Mock/FC, TGF-β/FCs, E-Mock/FCs, and E-TGF-β/FCs) were stained with FITC-conjugated mAbs against MUC1 and PE-conjugated mAbs against HLA-DR or matched isotype control IgG. After the cell aggregations were gated out [17], FC was determined by FACS analysis, where the fused cells were identified as MUC1^+^HLA-DR^+^. The numbers of events in each region were shown. (B) MFI of HLA-DR in double-positive populations (MUC1^+^HLA-DR^+^) of all four types of FC preparations (n = 4) was analyzed. (C) Percentage of cells positive for both MUC1 and HLA-DR in all four types of FC preparations (n = 4) were analyzed. The results are expressed as the mean ± SD. ***P<0.001; **P<0.01; *P<0.05.

To examine the effects of multiple immune-suppressive cytokines secreted by whole tumor cells on FC phenotypes, we developed four types of FC preparations by alternating tumor cells as fusion partners. Ethanol-treated or untreated tumor cells were successfully fused with TLRs-DCs (Fig. 4A). The fusion efficiency was determined by the percentage of MUC1 and HLA-DR double-stained cells (Fig. 4B). Although TGF-β/FCs showed lower MFI levels of HLA-DR on a per fusion basis, as compared with Mock/FCs (Fig. 4B). However, E-TGF-β/FCs exhibited improved fusion efficiency and up-regulation of MFI levels of HLA-DR, compared with TGF-β/FCs (Fig. 4B and C).

### Effects of Immune-suppressive Cytokines Secreted by Tumor Cells on FC Functions

**Figure 5:**
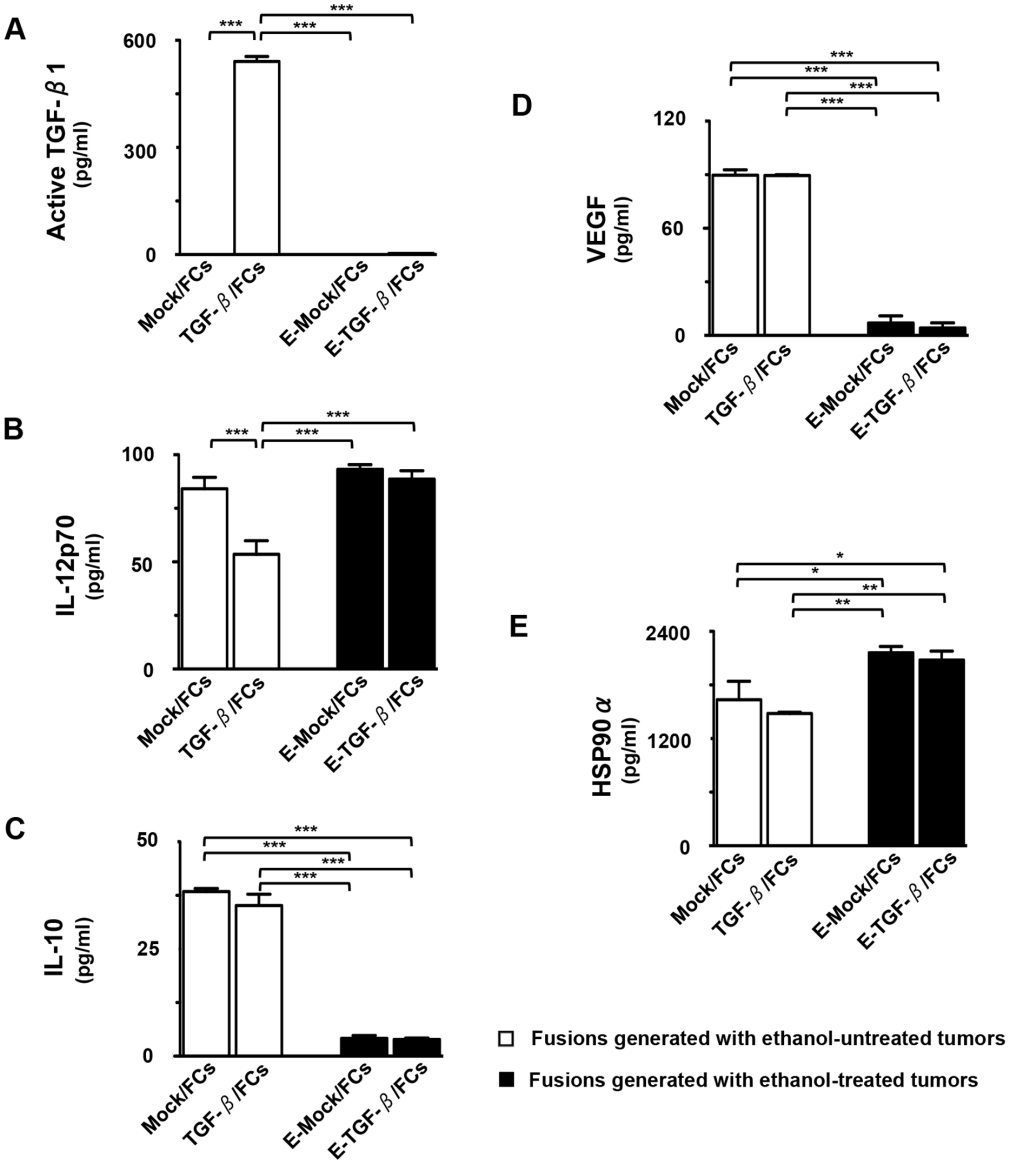
Functional characterization of FC preparations. The mean concentrations of active TGF-β1 (A), IL-12p70 (B), IL-10 (C), VEGF (D), and HSP90α (E) derived from four types of FC preparations (Mock/FCs, TGF-β/FCs, E-Mock/FCs, and E-TGF-β/FCs) (n = 4) were analyzed by ELISA. The background cytokine levels in conditioned medium were subtracted from each sample. The results are expressed as the mean ± SD. ***P<0.001; **P<0.01; *P<0.05.

To assess the effects of multiple immune-suppressive cytokines secreted by whole tumor cells on FC functions, the production of active TGF-β1, IL-12p70, IL-10, VEGF, and HSP90α in four types of FC preparations was examined. As expected, the active form of TGF-β1 was secreted at higher levels in TGF-β/FCs, compared with Mock/FCs (Fig. 5A). However, the levels of active TGF-β1 production in E-TGF-β/FCs were significantly reduced compared to TGF-β/FCs (Fig. 5A). On the other hand, high levels of IL-12p70 production were observed in Mock/FCs, compared to that was obtained in TGF-β/FCs (Fig. 5B). Importantly, recovery of IL-12p70 production was observed in E-TGF-β/FCs (Fig. 5B), suggesting the active form of TGF-β1 suppressed the function of DCs in the fused cell complex, and thus inhibiting IL-12p70 production. Moreover, the production of IL-10 and VEGF in E-Mock/FCs as well as E-TGF-β/FCs was also inhibited compared to Mock/FCs or TGF-β/FCs (Fig. 5C and D). Interestingly, E-Mock/FCs as well as E-TGF-β/FCs exhibited higher HSP90α production, compared to Mock/FCs or TGF-β/FCs (Fig. 5E). Moreover, HSP90α production was increased after fusion of TLRs-DCs and ethanol-treated tumor cells, compared with an unfused mixture of both (data not shown). In addition, there was no difference in the production of active TGF-β1, IL-12p70, IL-10, VEGF, and HSP90α between FC preparations generated with PANC1 or PANC/Mock (data not shown). Collectively, these results indicate that FC preparations generated with ethanol-treated tumor cells and TLRs-DCs inhibit the production of multiple immune-suppressive cytokines and up-regulate the production of IL-12p70 and HSP90α.

### T Cell Activation by FC Preparations

**Figure 6:**
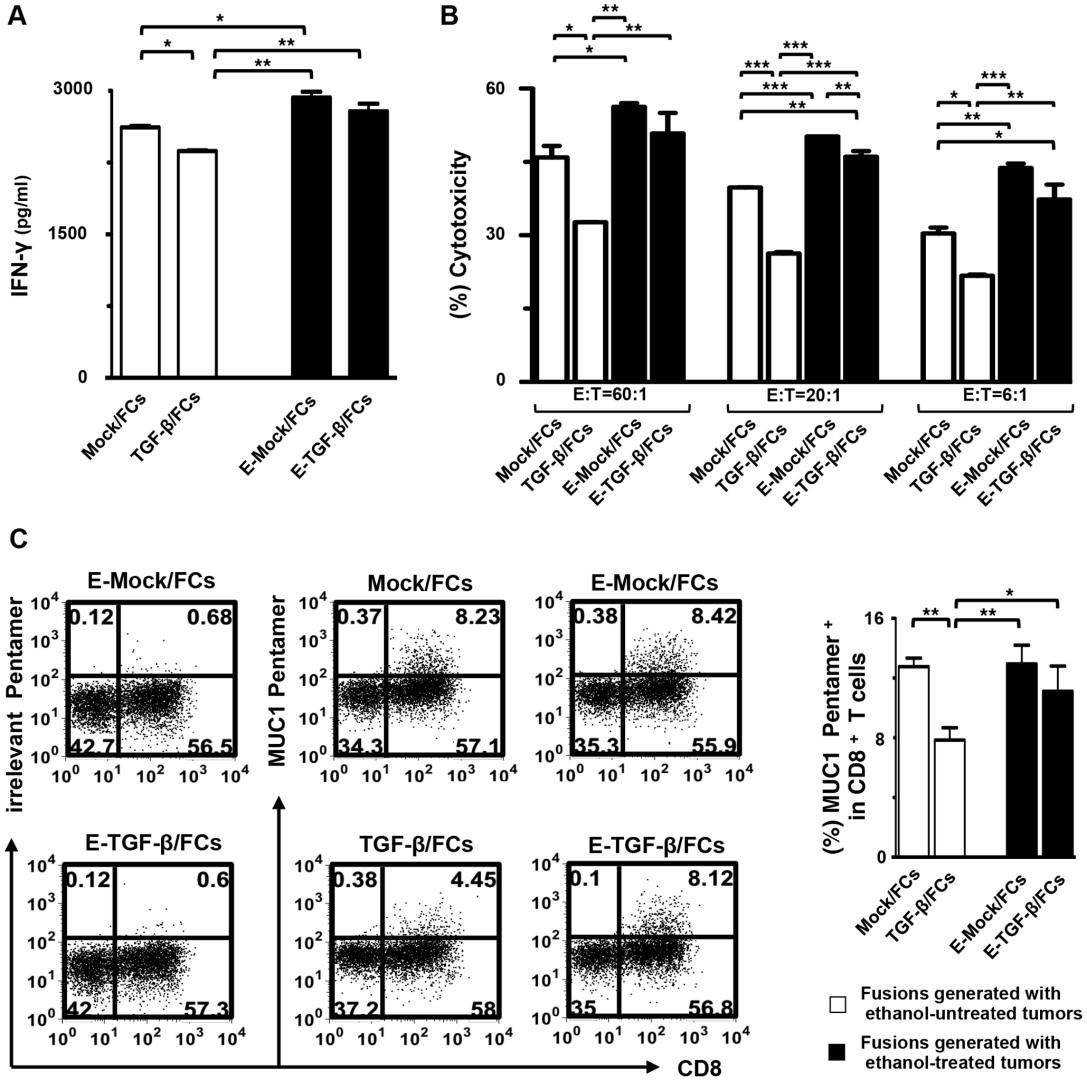
T cell stimulation by FC preparations. (A) T cells (1×10^6^/mL) (n = 3) were stimulated with four types of FC preparations (Mock/FCs, TGF-β/FCs, E-Mock/FCs, and E-TGF-β/FCs) in the absence of rhIL-2 for 2 days and IFN-γ-production was immediately analyzed by ELISA. The background cytokine levels in conditioned medium were subtracted from each sample. (B) T cells (n = 3) were stimulated with the same number of fused cells that coexpressed both MUC1 and HLA-DR in all four types of FC preparations in the same set of experiments. Stimulated T cells were incubated with PKH-26-labeled PANC-1 cells at the indicated E:T ratios (60∶1, 20∶1, 6∶1) for cytotoxicity assays. Percentage of cytotoxicity (mean ± SD) was determined by flow cytometry CTL assays. (C) T cells (HLA-A2^+^) (n = 3) stimulated by each types of FCs (HLA-A2^+^) were purified through nylon wool and stained with FITC-conjugated mAbs against CD8 and PE-conjugated pentamer against HLA-A2/MUC1 or irrelevant IgG (left panel). T cell populations were gated based on their forward- *vs.* side-scatter profile. The percentage of CD8^+^ T cells reactive to MUC1 among the whole CD8^+^ T cell population was shown as a percentage of the double-positive population (pentamer^+^CD8^+^) in the total CD8^+^ T cells (right panel). The results are expressed as the mean ± SD. ***P<0.001; **P<0.01; *P<0.05.

Although IFN-γ production by Mock/FCs was significantly higher compared to TGF-β/FCs, IFN-γ production by E-TGF-β/FCs as well as E-Mock/FCs was significantly increased compared to TGF-β/FCs or Mock/FCs (Fig. 6A). In contrast, there was little, if any, IFN-γ production by T cells cocultured with TLRs-DCs mixed with ethanol-treated tumor cells (data not shown). The low levels of IL-10 production by T cells stimulated by FC preparations did not impair the production of IFN-γ (data not shown).

### Augmentation of MUC1-specific CTLs by FCs Generated with Ethanol-treated Tumor Cells

The induction CTL activity against PANC-1 target cells was significantly reduced by TGF-β/FCs compared to Mock/FCs (Fig. 6B). However, T cells stimulated by E-TGF-β/FCs consistently exhibited higher CTL activity that preferentially lysed PANC-1 cells, compared with TGF-β/FCs (Fig. 6B). These results suggest that the tumor-derived immune-suppressive cytokines including TGF-β1 reduce the efficacy of FC vaccines *in vitro*. Moreover, the lysis was inhibited by pre-incubation of PANC-1 target cells with an anti-HLA-A2 mAb (data not shown), indicating restriction by HLA-A2. MUC1-specific and HLA-A2 restrictive CTL responses were also confirmed by pentamer staining. An increased percentage of MUC1-specific CD8^+^ T cells in the whole CD8^+^ T cell population was observed in the E-Mock/FCs, compared with TGF-β/FCs (Fig. 6C). In contrast, CTLs specific for MUC1 were not detected in T cells stimulated by an unfused mixture of DCs and tumor cells, whether treated with ethanol or not (data not shown). In addition, there was no difference in CTL activity against PANC-1 target cells induced by FCs generated with PANC/Mock or PANC-1 cells (data not shown). Together, these findings suggest that augmentation of CTL activity specific for MUC1 and restricted by HLA-A2 is generated by fusions of ethanol-treated tumor cells and TLRs-DCs *in vitro*.

## Discussion

This study demonstrate that *in vitro* antitumor immunity is synergistically augmented when FC preparations are accompanied by both ethanol-treated tumor cells and dual TLRs-stimulated DCs through TGF-β1 blockade and IL-12p70 production.

Multiple immune-suppressive cytokines inhibit the initiation of functional CTL responses [20], [21]. In particular, TGF-β1 has a critical role in immune-suppressive mechanisms such as reducing the number and function of circulating DCs [22], inactivation of CTLs [23], and generation of Tregs [24]. Thus, several strategies to target immune-suppressing signaling from tumor cells for whole tumor cell-based cancer vaccines have been developed using neutralizing antibodies [25], small molecular inhibitors [26], specific small interfering RNAs (siRNAs) [27], or expression of a soluble TGF-β receptor by tumor cells [28]. However, the complexity of all these methods may limit its use, especially in clinical settings. Therefore, we attempted to prepare immunogenic tumor cells for FC-based cancer vaccines that adhered to GMP processes. To ensure the delivery of a “cell drug” that is safe, reproducible, and efficient, we first established FCS-independent tumor cells that could grow with human PPF [16], as FCS has a possible risk of infection with pathogens of prion diseases for clinical use. Next, we demonstrated that the production of active TGF-β1, IL-10 and VEGR from tumor cells was significantly inhibited by a simple treatment of pharmaceutical grade ethanol without the down-regulation of MHC class I and the MUC1 tumor-associated antigen. Induction of apoptosis and necrosis by ethanol-treatment may be associated with decreased production of immune-suppressive cytokines from tumor cells. The ethanol-treatment is an extremely simple and rapid method, thus it may be suitable for the quick production of tumor cells with decreased production of immune-suppressive cytokines. One disadvantage of using ethanol-treated tumor cells is that it is necessary to perform a dose-response test to evaluate the optimal conditions of ethanol-treatment for each type of tumor cell. The alternative strategy of using ethanol-treated tumor cells for FC-based cancer vaccines may have some biological benefits as follows: (1) improved sterility and safety of tumor cell-based vaccines; (2) the molecular profile of TAAs can be better preserved in ethanol-treated cells, which retain sufficient structural integrity to directly prime T cells [29], [30]; (3) the denaturation or modification of TAAs by ethanol-treatment exposes a more potent immunogenic moiety that is normally hidden [31]; (4) protein aggregation by ethanol-treatment exposes hydrophobic portions of the antigens that tend to be strong immune stimulators [32], [33]; (5) ethanol, at concentrations that affect tumor growth rates, is also a potent inducer of HSPs [34], [35]. HSPs exposed by tumor cells can be recognized by TLR4 expressed on DCs, which facilitates activation, intracellular antigen-processing and -presentation in DCs [36], [37]; and (6) CRT and HMGB1 exposed on ethanol-treated tumor cells serve “eat-me” signals [38], [39]. It has been reported that only tumor cells that undergo immunogenic apoptosis ectopically expose the Ca^2+^-binding chaperone, CRT, that allows TAAs to traffic to the antigen-presenting compartment in DCs [38], [39]. Moreover, HMGB1 passively released from tumor cells interact with TLR4 on DCs, and result in the stimulation, antigen-processing and -presentation in DCs [39]–[41].

Recently, we demonstrated that the activation of FCs by dual-administered TLR agonists is more effective in comparison to those single activated or un-activated FCs, however, immune-suppressive cytokines such as TGF-β1 secreted from whole tumor cells inhibited the activation of FCs [42]. The results shown here that improved immunogenicity of whole tumor cells by ethanol-treatment led us to speculate that FCs generated with DCs stimulated with dual TLR agonists and ethanol-treated tumor cells would be more effective than conventional FCs. Indeed, FCs generated with ethanol-treated tumor cells sustained high levels of IL-12p70 production. Moreover, in FCs generated with ethanol-treated tumor cells, IL-10 was lower concentration and HSP90α was higher than those in fusion generated with ethanol-untreated tumors. The tendency was same, compared to tumor cells alone. However, fusions produced higher levels of HSP90α, compared to an unfused mixture of TLRs-DCs and ethanol-treated tumor cells. During fusion-process, PEG-treatment may also induce HSP90α production by FCs. This is the reason, at least in part, why we observe the increased levels of HSP90α production of fusions. The transcriptional activation of a series of molecular chaperones such as HSP90α that can bind peptides derived from TAAs is a common response to cell stress [36], [37], including that induced by ethanol and PEG, which might associated with enhanced CTL responses.

The fusion strategy is also associated with induction of efficient CTL responses. We used PEG to facilitate a larger initial contact surface between the tumor cells and DCs [42], [43]. Thus, tight contact between DCs producing high levels of IL-12p70 and tumor cells expressing CRF, HMGB1, and HSP90α with inhibited production of immune-suppressive cytokines during the fusion process may act as essential recognition and danger/alarm signals to FCs, leading to increased fusion efficiency and function to induce efficient antigen-specific CTLs *in vitro*. Our findings were also consistent with previous reports that combined TLR2/4 agonists cooperate efficiently with HSPs and HMGB1 in activating DCs to induce augmented CTL responses [39], [43].

In conclusion, this study demonstrates that augmented CTL responses are primed by FC preparations generated with ethanol-treated whole tumor cells and combined TLRs-stimulated DCs *in vitro*. However, it is still unclear which specific treatments, such as cytotoxic chemotherapeutic drugs, ionizing irradiation, and chemical agents, lead to significant immunogenic tumor cell death and which combinations of TLR agonists have the greatest synergy for cancer vaccines. An improved understanding of these issues may facilitate the integration of immunogenic tumor cells and activated DCs for FC-based cancer vaccines.
